# Epigenetic Regulation of Immune Dysfunction in Chronic Prostatitis/Chronic Pelvic Pain Syndrome

**DOI:** 10.1002/pros.70191

**Published:** 2026-05-04

**Authors:** Praveen Thumbikat, Goutham Pattabiraman, Farzaneh Sharifzad, Yongyong Yang, Zhiqiang Liu, Catherine V. Osborn, Stephen F. Murphy, Qi Cao, Anthony J. Schaeffer

**Affiliations:** ^1^ Department of Urology, Feinberg School of Medicine Northwestern University Chicago Illinois USA

**Keywords:** chronic pain, DNA methylation, epigenetics, FoxP3, IL10, immune dysregulation, pelvic pain, UCPPS

## Abstract

**Background:**

Chronic prostatitis/chronic pelvic pain syndrome (CP/CPPS) is a prevalent and debilitating condition with unclear etiology. Increasing evidence implicates immune dysregulation, yet the molecular mechanisms underlying impaired immune regulation remain poorly defined. This study investigated the role of altered immune responses within PBMC populations and DNA methylation in CP/CPPS pathogenesis.

**Methods:**

Post‐prostatic massage urine samples designated as Voided Bladder 3 (VB3) urine from CP/CPPS patients and healthy controls were analyzed for CD4^+^ T cell markers and lineage‐defining transcription factors. DNA methylation profiling of peripheral blood mononuclear cells (PBMCs) and purified CD4^+^ T cells was performed using targeted methylation arrays. Functional assays evaluated IL10 production following lipopolysaccharide (LPS) stimulation, with or without azacitidine (AZA), a DNA methyltransferase inhibitor that reverses methylation‐dependent gene silencing. In vivo relevance was assessed using the experimental autoimmune prostatitis (EAP) mouse model.

**Results:**

VB3 samples from CP/CPPS patients demonstrated elevated CD4‐associated transcripts and increased expression of immune‐related transcription factors including *RORγT*. DNA methylation analysis identified differences in methylation across immune‐regulatory loci including *IL10, FOXP3, CD274, ITGAL*, and *TNF‐α*. PBMCs from patients exhibited diminished IL10 secretion in response to LPS, which was restored by AZA treatment. In the EAP model, recombinant IL10 administration failed to attenuate pelvic allodynia, whereas AZA significantly reduced pain sensitivity.

**Conclusions:**

CP/CPPS is associated with epigenetic alterations in immune regulatory genes that may influence inflammatory responses. Pharmacologic inhibition of DNA methylation enhanced IL10 responses in vitro and reduced pain behaviors in vivo, supporting demethylation therapy as a potential strategy for treating chronic prostatic inflammation and pelvic pain.

## 1 Introduction

Chronic prostatitis/chronic pelvic pain syndrome (CP/CPPS) is a common condition within the spectrum of urologic chronic pelvic pain syndrome (UCPPS), diagnosed exclusively in adult men [1, 2, 3]. Despite its high prevalence and impact on quality of life, the etiology and pathogenesis of CP/CPPS remain poorly understood. The condition is typically diagnosed by exclusion and is characterized by persistent pelvic pain, often accompanied by lower urinary tract symptoms and sexual dysfunction.

Over the past two decades, multiple hypotheses have been proposed to explain CP/CPPS pathophysiology, including autoimmunity, hormonal imbalances, and pelvic floor dysfunction [4]. More recently, neuroimaging studies have identified alterations in neuronal density and functional connectivity in the brains of CP/CPPS patients, although it remains unclear whether these changes are primary drivers or secondary adaptations to pathology elsewhere in the body [5].

Experimental studies in animal models have demonstrated that immune responses directed against the prostate are sufficient to induce hallmark symptoms of pelvic pain and voiding dysfunction [6, 7, 8, 9, 10, 11]. Although the precise immune triggers vary, some involving IFN‐γ–driven pathways [12], others, including work from our group, implicating IL17–mediated responses [7, 9, 12, 13], a unifying feature across models is the activation of adaptive immunity, particularly T cell‐mediated inflammation. In chronic immune‐mediated diseases, a growing body of evidence highlights the role of epigenetic modifications in shaping immune susceptibility. Epigenetic mechanisms, including chromatin remodeling, noncoding RNAs, and DNA methylation, control gene expression without altering the underlying DNA sequence and are responsive to developmental, metabolic, and environmental inputs [14, 15]. Among these, DNA methylation is the most extensively studied and involves covalent modification of cytosine residues within CpG islands, catalyzed by DNA methyltransferases (DNMTs) and interpreted by methyl‐CpG‐binding proteins [16, 17, 18, 19]. Aberrant DNA methylation patterns have been implicated in a wide array of pathological states by disrupting immune tolerance or promoting inflammatory gene expression [20]. Epigenome‐wide association studies (EWAS) have linked methylation changes to environmental exposures such as chemical toxins and smoking, as well as to diseases characterized by chronic inflammation, including inflammatory bowel disease, Alzheimer's disease, cancer, and diabetes [21, 22, 23, 24]. Across chronic pain conditions, DNA methylation has emerged as a key and durable regulator of gene programs that sustain pain long after the initiating insult has resolved [25]. Models of peripheral nerve injury and other persistent pain show coordinated methylation shifts at promoters controlling neurotransmission, intracellular signaling, and immune pathways, with methylation often inversely tracking mRNA expression and correlating with pain intensity [26]. Critically, T‐cell methylation signatures substantially overlap with pain‐linked patterns in the prefrontal cortex, and compact T‐cell methylation panels can classify neuropathic pain and predict mechanical hypersensitivity [26]. These findings position peripheral immune cells as minimally invasive windows into central pain biology while highlighting methylation‐encoded states as actionable targets for disease modification [25, 26].

Given these observations, we hypothesized that aberrant DNA methylation contributes to the immune imbalance observed in CP/CPPS. We further posited that these changes disproportionately affect regulatory immune pathways. This is based on the observation that multiple distinct proinflammatory mechanisms—including IL17 and IFN‐γ can induce pelvic pain in animal models, suggesting that failure of regulatory restraint, rather than the nature of the initial immune stimulus, may be a common determinant of chronic disease persistence.

## 2 Methods

### 2.1 Study Population and Specimen Collection

Men ≥ 18 years old who were healthy or had a prior diagnosis of Category III CP/CPPS were recruited through the Northwestern University Feinberg School of Medicine Department of Urology via physician referral, advertisements, and ClinicalTrials.gov outreach (NCT01676857, NCT03167216, NCT05185180). Eligibility for CP/CPPS patients included the presence of pelvic pain or discomfort for ≥ 3 months within the preceding 6 months and a National Institutes of Health‑Chronic Prostatitis Symptom Index (NIH‑CPSI) total score ≥ 12/43 at screening. Dr A. Schaeffer and colleagues collected questionnaire data, Voided Bladder 3 (VB3) urine, and blood samples at the Northwestern Memorial Hospital outpatient urology clinic, Chicago, IL. The Northwestern University Institutional Review Board (Panel D) approved the protocols (IRB STU00030121, STU00202831, and STU00215831), and all participants provided written informed consent. In comparison of age distribution of different CP/CPPS and control cohorts used for analyses; no statistically significant differences were observed (Table 1). Subjects were not analyzed separately based on CP/CPPS subtype. The 2025 AUA Guideline for Male Chronic Pelvic Pain (CP/CPPS) notes that although the condition is historically divided into inflammatory (NIH Category IIIA) and non‐inflammatory (NIH Category IIIB) subtypes, “there is no evidence that patients in the two subgroups exhibit different symptoms or respond differently to therapy” [27]. Accordingly, subtype stratification was not performed for the present analyses. Medication status was not controlled for in this study, as patients were recruited during routine clinical care and treatment exposure was heterogeneous. These variables represent potential confounders and are acknowledged as limitations of the present study.

**Table 1:** Age distribution of CP/CPPS patients and controls across study cohorts.

	CP/CPPS patients (*n*)	Age (mean ± SD), yrs	Controls (*n*)	Age (mean ± SD), yrs	*p*
VB3 cell analysis	23	44.4 ± 15.0	4	36.3 ± 12.1	0.285
PBMC epigenetic analysis	10	44.6 ± 12.8	10	33.9 ± 10.7	0.056
CD4 epigenetic analysis	9	50.3 ± 15.4	8	42.9 ± 7.7	0.22

*Note:* Values represent mean ± SD. Age distributions were compared using Welch's two‐sample *t*‐tests.

Post‐prostatic massage urine samples that are clinically considered to be enriched for prostate‐derived secretions were centrifuged (300*g*, 4 min, 4°C); supernatants were stored at −80°C, and cell pellets were resuspended and used for RNA extraction. Peripheral blood was drawn into BD Vacutainer CPT tubes preloaded with Ficoll and processed within 2 h by centrifugation (1800*g*, 20 min, room temperature) to isolate the peripheral blood mononuclear cell (PBMC) layer. To cryopreserve PBMCs, the cells were resuspended gently at 0.5–10 × 10^6^ cells/mL in 20% DMSO in FBS at a ratio of 1:1 for a final concentration of 10% DMSO and 90% FBS. Cells were cooled to extremely low temperatures, then stored at liquid nitrogen temperatures (below −135°C) until needed. Storage and handling conditions were standardized between controls and CP/CPPS patients. Due to limitations in matched sample availability, VB3 transcript analyses, PBMC methylation studies, and CD4^+^ T‐cell methylation analyses were performed on partially overlapping patient cohorts.

### 2.2 Quantitative Real‐Time PCR (qPCR)

Total RNA was extracted from the VB3 cells with TRIzol reagent (Invitrogen) and reverse‑transcribed with qScript cDNA SuperMix (QuantaBio) following the manufacturers' protocols. qRT‑PCR reactions were prepared with PerfeCTa SYBR Green SuperMix (QuantaBio) and run on a CFX Connect Real‑Time PCR System (Bio‑Rad). Appropriate primers that specifically amplified human *CD4* (forward sequence‐CCTCCTGCTTTTCATTGGGCTAG, reverse sequence ‐TGAGGACACTGGCAGGTCTTCT Origene), *T‐bet, GATA‐3, RORγT*, and *FOXP3* were selected based on previously published and validated primer sets [28]. *GAPDH* was measured as a housekeeping gene for normalization. Relative transcript levels were calculated by the 2^−ΔΔCt^ method normalized to *GAPDH* expression, with subsequent data visualization and statistical analysis performed in GraphPad Prism (GraphPad Software). In VB3 analyses, expression of selected markers was also considered relative to *CD4* transcript levels to estimate immune‐associated expression within mixed samples.

### 2.3 CD4 T Cell Extraction

PBMCs were cryopreserved in standard freezing medium and stored in liquid nitrogen until use. For enrichment of CD4^+^ T cells, PBMCs were thawed, washed, and assayed for adequate cell viability. CD4^+^ T cells were enriched using the EasySep Human CD4^+^ T Cell Isolation Kit (STEMCELL Technologies) according to manufacturer instructions. Expected purity exceeds 90% based on kit validation data, and pilot experiments using control PBMC samples demonstrated a typical purity range of approximately 90%–95% by flow cytometry. Purity of the isolated CD4^+^ T‐cell population was verified by flow cytometry in pilot experiments using control PBMC samples. Routine verification was not performed for each sample due to limited sample availability. Purified cells were then pelleted and shipped on dry ice to EpigenDx (Hopkinton, MA) for methylation analysis.

### 2.4 DNA Methylation Studies

DNA methylation profiling was performed using targeted next‐generation bisulfite sequencing (tNGBS) with custom panels NGS070V3 and NGS178 (EpigenDx, Table 2), designed to examine regulatory regions of immune‐related genes including *FOXP3*, *IL10*, *IL17*, *IFNγ*, and *ITGAL*. Genomic DNA isolated from cell pellets was lysed using M‐digestion buffer (2× final concentration) with 5–10 µL protease K (20 mg/mL) and incubated at 65°C for ≥ 2 h. Bisulfite conversion was performed on 20 µL of extracted DNA using the EZ‐96 DNA Methylation‐Direct Kit (Zymo Research), with modified conditions, and DNA was eluted in 46 µL of elution buffer. Bisulfite‐modified DNA was amplified using multiplex or simplex PCR reactions containing 0.5 U HotStarTaq DNA polymerase (Qiagen), 0.2 µM primers, and 3 µL template DNA in a 20 µL reaction. PCR cycling conditions were 95°C for 15 min; 45 cycles of 95°C for 30 s, annealing temperature (Ta) for 30 s, and 68°C for 30 s; followed by 68°C for 5 min. PCR products were verified using the QIAxcel Advanced System, pooled per sample, and purified using QIAquick PCR purification columns or plates. Sequencing libraries were prepared using a custom EpigenDx protocol, purified with AMPure XP beads, barcoded, and pooled equimolarly. Template preparation and enrichment were performed on the Ion Chef system, followed by sequencing on the Ion S5 platform using Ion 530 chips.

**Table 2:** Gene list for targeted DNA methylation assays (panels NGS070V3 and NGS178).

Gene	Assay(s)	CpGs
S100A6	ADS1798	8
CTLA4	ADS3074B; ASY1526	5; 6
IL4	ADS1623R	4
IL2	ADS740	1
IL2RA	ASY1521	4
IL2RB	ASY1523; ASY1522	4; 4
PDCD1	ADS2509; ADS2744	4; 5
PDL2	ADS5320; ADS5321; ADS5323	3; 2; 1
CD274	ADS1929	5
TNF	ADS711	3
TNFRSF18	ADS4562	12
TNFRSF25	ADS319	9
ETS1	ADS3144	3
RUNX1	ADS3030	5
FOXP3	ADS6645A; ADS783; ASY1524; ASY1352re; ADS8534A	8; 11; 5; 3; 2
IKZF2	ADS4563	3
IKZF4	ADS4565	2
TOLLIP	ADS3481	5
CD3D/CD3G	ADS3513	3
CDKN1C	ADS1787	11
ITGAL	ASY3779; ADS548B; ASY1735; ASY3777	2; 5; 3; 2
IL10	ADS1065re; ADS6734re; ADS8533re; ADS1064	2; 1; 5; 4
IFNG	ASY569re; ASY568re	3; 2
IL17	ADS2466A; ADS2467re; ADS2345B	1; 2; 3

For downstream analysis, FASTQ files were aligned to a bisulfite‐converted reference genome using Bismark (v0.12.2) with the Bowtie2 algorithm (v2.2.3). CpG methylation levels were quantified as the ratio of methylated to total reads at each site. Assay design incorporated stringent filtering to exclude regions with repetitive elements, low sequence complexity, high thymidine content, or excessive CpG density to ensure specificity and analytical robustness.

### 2.5 Cell Culture Studies

PBMCs were thawed in a 37°C water bath and slowly diluted with prewarmed RPMI 1640 medium supplemented with 10% fetal bovine serum. After centrifugation at 300*g* for 5 min, the cells were resuspended in fresh culture medium and incubated at 37°C with 5% CO_2_ for 1 h to allow recovery. For in vitro inflammation studies, 0.5 × 10^5^ cells/well in 6‐well plates were stimulated with 1 μg/mL/well of LPS (Sigma); and incubated for 24 h at 37°C, 5% CO_2_. Supernatants were collected by centrifugation at 300*g* for 5 min and stored at −80°C. IL10 levels were quantified using the Quantikine Human IL10 ELISA Kit (R&D Systems, Catalog #D1000B) according to the manufacturer's instructions. For in vitro methylation inhibition studies, PBMC's were treated with 1 μM DNMT inhibitor, azacitidine (AZA) for 48 or 96 h followed by isolation of total RNA and qRT‑PCR for *IL10* expression.

### 2.6 Therapeutic Studies in Experimental Autoimmune Prostatitis (EAP)

CP/CPPS was modeled using mice with experimental autoimmune prostatitis (EAP) as previously described [11]. Animal experiments procedures were approved by the Northwestern University Animal Care and Use Committee. Briefly, male NOD/ShiLtJ mice (5–7 weeks old) were immunized subcutaneously with 100 μg of rat prostate antigen emulsified in TiterMax Gold adjuvant (1:1 ratio, GA 30093) (Day 0) and allowed to develop autoimmune‐mediated prostate inflammation over 28 days. Behavioral assessment of changes in mechanical allodynia of the pelvic region was quantified from von Frey filament testing on Days 0, 7, 14, 21, and 28 postimmunization. To evaluate the therapeutic effects of IL10, from Day 22 to Day 27, mice received daily intraperitoneal injections of 1 μg recombinant mouse IL10 in 100 μL PBS while controls received vehicle injections (sterile PBS). For AZA therapeutic assessment, male C57BL/6J mice (5–7) weeks of age were allowed to develop EAP and treatment was initiated at 28 days after EAP by intraperitoneal injection of 5‐AZA (A3666, Sigma) at 2.5 mg/kg. Injections were repeated daily for 7 days. Behavioral assessment of changes in mechanical allodynia of the pelvic region was quantified from von Frey filament testing at the onset of EAP, and at the beginning and end of the treatment regimen.

### 2.7 Statistical Analyses

Statistical analyses were performed using GraphPad Prism (GraphPad Software, San Diego, CA, USA). Statistical tests utilized in each experiment are indicated in figure legends (in most figures, each dot represents individual patients/animals). Data are presented as mean ± standard error of the mean unless otherwise indicated. *p < 0.05, **p < 0.01, ***p < 0.001, ****p < 0.0001.

## 3 Results

### 3.1 Immune‐Associated Transcript Signatures Detected in VB3 Urine Sediments From CP/CPPS Patients

**Figure 1:**
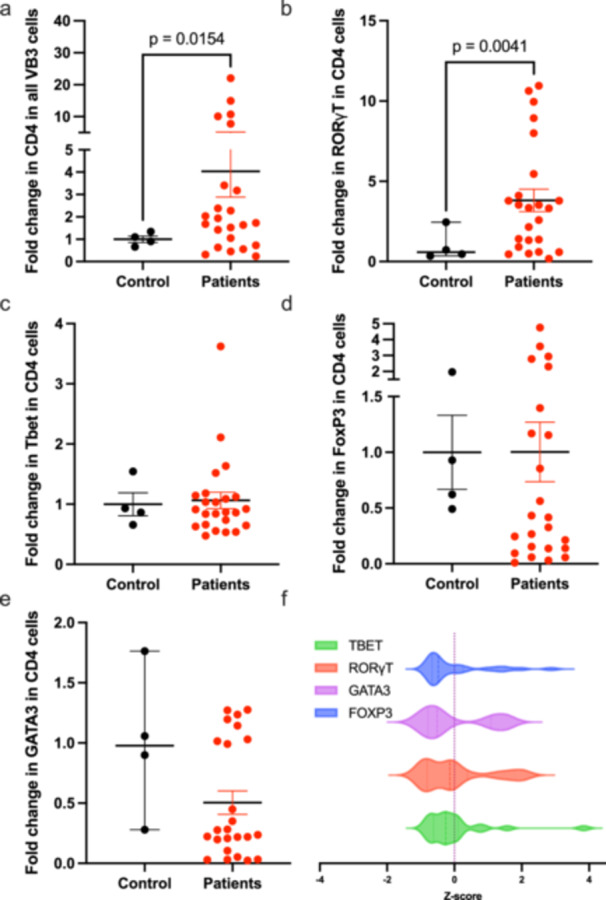
*CD4+* cells from VB3 urine demonstrate altered immune responses. Cells were isolated from VB3 urine samples of CP/CPPS patients (*n* = 24) and healthy controls (*n* = 4) and analyzed by qRT‐PCR for genes associated with adaptive immune function. (a) *CD4* mRNA expression; (b–e) expression of key transcription factors: *TBET* (Th1), *RORγT* (Th17), *GATA3* (Th2), and *FOXP3* (Treg), transcript levels expressed relative to *CD4* transcript abundance as an estimate of T‐cell representation within mixed cellular samples. Statistical comparisons were performed using two‐tailed unpaired *t*‐tests; individual *p* values are shown. (f) *Z*‐scores were calculated as (*X* − mean)/standard deviation, where a *Z*‐score of 0 indicates a value equivalent to the control mean, and positive or negative scores reflect the magnitude of deviation. [Color figure can be viewed at wileyonlinelibrary.com]

To assess local adaptive immune responses, we profiled CD4^+^ T‐cell–associated transcripts in VB3 urine sediments from men with CP/CPPS (*n* = 24) and healthy controls (*n* = 4; NU IRB STU00030121). While the limited numbers of immune cells in VB3 sediment precluded analytical methods such as flow cytometry, qRT–PCR revealed a significant increase in *CD4* mRNA expression in CP/CPPS samples compared with controls (*p* = 0.0154; Figure 1a), consistent with increased abundance of CD4‐associated transcripts in VB3 sediments. To further explore immune‐associated transcriptional signatures, we studied expression of lineage‐defining transcription factors—*TBET* (Th1), *RORγT* (Th17), *GATA3* (Th2), and *FOXP3* (Treg)—normalized to CD4 expression. Among these, *RORγT* was significantly upregulated in patients (*p* = 0.0041; Figure 1b–e), whereas *TBET, GATA3*, and *FOXP3* showed no consistent group‐level differences. Notably, expression profiles displayed marked interindividual heterogeneity. *Z*‐score analysis revealed distinct patient subsets with elevated *RORγT* and *GATA3* and reduced *FOXP3* and *TBET* expression relative to the control mean (Figure 1f). These findings suggest heterogeneous immune‐associated transcriptional signatures are detectable in VB3 sediments from CP/CPPS patients.

### 3.2 Aberrant DNA Methylation Signatures in PBMCs From CP/CPPS Patients

**Figure 2:**
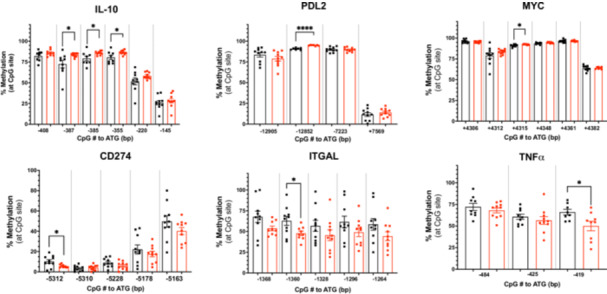
Aberrant DNA methylation signatures in PBMCs from CP/CPPS patients. Peripheral blood mononuclear cells (PBMCs) from a separate cohort of CP/CPPS patients (*n* = 10) and healthy controls (*n* = 10) were analyzed for DNA methylation changes in genes linked to FOXP3 expression and T‐regulatory function using the EpigenDx Human FOXP3 array. Methylation at multiple CpG sites was assessed and compared between groups. Statistical comparisons between groups were performed using two‐tailed unpaired *t*‐tests; *p < 0.05, ****p < 0.0001. [Color figure can be viewed at wileyonlinelibrary.com]

To determine whether systemic immune dysregulation in CP/CPPS is associated with epigenetic alterations, we analyzed PBMCs from CP/CPPS patients (*n* = 10) and age‐matched healthy controls (*n* = 10) using a targeted methylation array focused on regulatory immune genes. Multiple loci displayed significant differences in DNA methylation between patients and controls. The *IL10* promoter was hypermethylated at three CpG sites located at –387, –385, and –355 base pairs (bp) from the ATG start site (Figure 2). This is consistent with reduced expression of this anti‐inflammatory cytokine observed in previous studies [29]. Additional hypermethylation was detected in *PDL2* and *MYC*, whereas *CD274* (PD‐L1), *ITGAL* (LFA‐1), and *TNF*‐α loci were observed to be hypomethylated (Figure 2). Although the absolute methylation differences at individual CpG sites were modest, the overall pattern indicated consistent methylation differences across several immune‐related loci. These data support the presence of systemic epigenetic alterations in CP/CPPS that may contribute to the reported proinflammatory immune phenotype.

### 3.3 Targeted Methylation Analysis in Purified CD4^+^ T Cells

**Figure 3:**
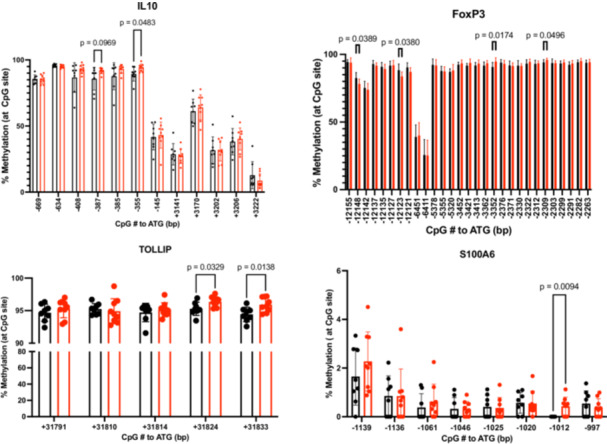
Targeted methylation analysis in purified CD4^+^ T cells. DNA methylation analysis was performed on purified CD4^+^ T cells isolated from the peripheral blood of CP/CPPS patients (*n* = 9) and age‐matched healthy controls (*n* = 8) using the EpigenDx Human FOXP3 array. Methylation at multiple CpG sites was assessed and compared between groups, with specific CpG sites identified relative to the ATG start site. Statistical comparisons were performed using two‐tailed unpaired *t*‐tests; individual *p* values are shown. [Color figure can be viewed at wileyonlinelibrary.com]

Given the central role of CD4^+^ T cells in models of CP/CPPS pathogenesis [10, 11], we next examined whether the DNA methylation alterations observed in PBMCs were associated with this subset. Purified CD4^+^ T cells from a separate cohort of CP/CPPS patients (*n* = 9) and age‐matched healthy controls (*n* = 8) were analyzed using a targeted methylation array focused on regulatory immune loci. Consistent with the prior PBMC findings, modest increases in *IL10* promoter methylation were observed in CD4^+^ T cells from CP/CPPS patients (Figure 3). In addition, promoter hypermethylation was observed in the *Foxp3* gene locus in the conserved noncoding sequences (CNSs) that are key functional enhancer elements for induction and stabilization of Foxp3 expression [30]. Hypermethylation was observed in CNS1 that is known to contain the transforming growth factor‐β (TGF‐β) response element, which contributes to extrathymic Treg cell generation and mucosal immune tolerance [31] and in CNS2 known to be responsible for the stability of Foxp3 in response to T cell receptor (TCR) stimulation [32]. These *FOXP3* changes were not evident in bulk PBMCs, suggesting that these regulatory lesions were masked in mixed immune cell populations. CD4^+^ T cells from patients also exhibited increased methylation at *TOLLIP* and *S100A6*, genes involved in innate immune and inflammatory regulation. These findings suggest that CD4^+^ T cells from CP/CPPS patients exhibit epigenetic differences at selected immune‐regulatory loci.

### 3.4 Functional Correlates of Increased *IL10* Promoter Methylation

**Figure 4:**
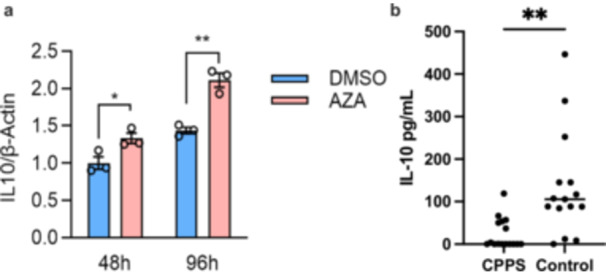
Functional consequences of increase in IL10 promoter methylation. (a) PBMCs from CP/CPPS patients were treated with 1 μM AZA for 48 or 96 h to inhibit DNA methylation, followed by qRT‐PCR for *IL10* expression. (b) PBMCs from CP/CPPS patients were stimulated in vitro with LPS (1 μg/mL, 24 h), and IL10 levels in supernatants were quantified by ELISA. Statistical comparisons were performed using two‐tailed unpaired *t*‐tests; *p < 0.05, **p < 0.01. [Color figure can be viewed at wileyonlinelibrary.com]

To determine whether *IL10* expression is epigenetically regulated, PBMCs from healthy donors were treated with the DNMT inhibitor AZA. AZA treatment produced a time‐dependent increase in *IL10* mRNA expression, suggesting that *IL10* expression may be influenced by pharmacologic inhibition of DNA methylation (Figure 4a). Because IL10 is typically induced by proinflammatory stimuli to dampen ongoing immune responses, we next assessed the functional impact of the increase in *IL10* promoter methylation observed in CP/CPPS patients. PBMCs from healthy controls responded to lipopolysaccharide (LPS) stimulation by producing robust levels of IL10, whereas PBMCs from CP/CPPS patients showed a markedly diminished IL10 response (Figure 4b). These findings suggest underlying differences in the ability to induced IL10 expression in response to inflammatory stimuli in mixed PBMC populations from CP/CPPS patients.

### 3.5 In Vivo Validation of Epigenetic Therapy in the EAP Model

**Figure 5:**
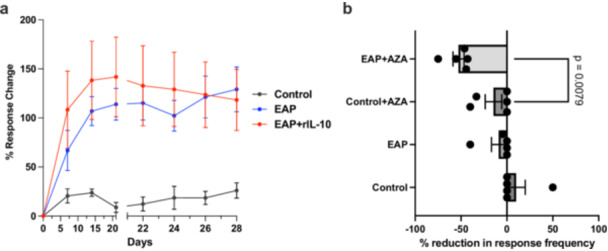
In vivo validation of epigenetic therapy in the EAP model. (a) In vivo therapeutic studies were conducted in experimental autoimmune prostatitis (EAP) mouse models. NOD/ShiLtJ mice (6–7/group) were immunized with rat prostate antigen and treated with recombinant mouse IL10 (1 μg/day, i.p., Days 22–27). Mechanical allodynia was assessed by von Frey filament testing. (b) In a separate EAP cohort, C57BL/6J mice (5/group) received 5‐AZA (2.5 mg/kg/day, i.p., Days 28–34), and mechanical allodynia was assessed before and after treatment. Statistical analyses were performed using ANOVA with multiple comparisons; individual *p* values are shown. [Color figure can be viewed at wileyonlinelibrary.com]

To determine whether restoring regulatory function through demethylation could alleviate pelvic pain in vivo, we employed the EAP model in male NOD/ShiLtJ mice, a well‐established murine model of CP/CPPS. Mice were immunized with prostate antigen and allowed to develop chronic pelvic pain, quantified by mechanical allodynia using von Frey testing. Administration of recombinant IL10 beginning on Day 22 of disease did not alter pain response frequency (Figure 5a), indicating that cytokine supplementation alone was insufficient once regulatory defects were established. In separate experiments using C57BL6/J mice, we recapitulated the EAP model and demonstrated that treatment with the DNMT inhibitor AZA, initiated on Day 28 after EAP significantly reduced pelvic allodynia within 1 week of therapy (Figure 5b). These data provide proof‐of‐concept evidence that pharmacologic inhibition of DNA methylation may reduce pelvic pain behaviors in the EAP model and that epigenetic modulation may influence inflammatory pain responses.

## 4 Discussion

Emerging evidence indicates that epigenetic mechanisms, particularly DNA methylation, contribute to CP/CPPS pathogenesis. Prior studies identified hypermethylation of estrogen receptor genes (*ESR1/ESR2*) linked to elevated estradiol levels [33], sperm DNA fragmentation and altered protamine ratios [34], and differential neuroinflammatory gene expression in CP/CPPS cohorts [35]. However, the functional significance of epigenetic changes within immune cells remains underexplored. Our study addresses this by identifying immune‐related methylation alterations in *IL10* and *FOXP3* associated with CP/CPPS.

Our findings show increased *CD4* ^+^ expression and elevated *RORγT* in post‐prostatic massage urine, consistent with increased expression of *RORγT*‐associated transcripts and prior studies implicating IL17 in CP/CPPS pathogenesis [9]. We identified increased promoter methylation at multiple CpG sites in the *IL10* promoter, potentially influencing expression of this key anti‐inflammatory cytokine. This is supported by prior work showing lower frequency of IL10–producing genotypes in CP/CPPS patients (30.6% vs. 12.1%, *p* = 0.007), which correlated with inflammation and treatment response [29].

Hypomethylation of the *ITGAL* promoter suggests increased leukocyte adhesion capacity, while altered methylation in *PDL2*, *TNF‐α*, and *MYC* indicates a broader inflammatory activation profile. Interestingly, no *FOXP3* methylation change was detected in PBMCs; however, purified CD4^+^ T cells revealed hypermethylation at CNS1 and CNS2—regions critical for *FOXP3* expression and Treg stability.

We also observed consistent methylation changes in *TOLLIP* and *S100A6*, genes linked to innate immunity and inflammation. These patterns suggest distinct immune cell subsets contribute to CP/CPPS pathophysiology. Comparison of PBMCs and CD4^+^ T cells highlighted the importance of cell‐type resolution, as several inflammatory gene methylation changes appeared specific to non‐CD4^+^ populations. Both hypermethylation and hypomethylation at immune regulatory loci may reflect altered immune regulation rather than unidirectional suppression.

*IL10* expression increased following AZA treatment, suggesting that DNA methylation may influence its expression. Moreover, IL10 induction following LPS stimulation was blunted in CP/CPPS patients. In vivo, recombinant IL10 failed to reverse pelvic pain in the mouse model, whereas AZA significantly improved pain sensitivity, suggesting that there is potential therapeutic benefit in targeting methylation over cytokine supplementation. These findings should be interpreted as associative, as methylation status was not directly measured in the same samples before and after AZA treatment.

These findings align with studies in other autoimmune diseases. *IL10* promoter hypermethylation has been linked to reduced cytokine expression in a number of autoimmune diseases including SLE and RA [36]. Similarly, *FOXP3* hypermethylation at the TSDR (Treg‐specific demethylated region) impairs Treg function in RA and other immune and nonimmune disorders [37, 38]. Our findings support the view that CP/CPPS shares immune‐epigenetic features with other chronic inflammatory disorders and may reflect systemic dysregulation rather than being limited to the pelvic region. Recent studies, including the MAPP Research Network, have emphasized the concept of “widespreadness,” showing that a subset of UCPPS patients experience multisite pain and overlap with other chronic pain and autoimmune disorders such as fibromyalgia, irritable bowel syndrome, and chronic fatigue syndrome [39, 40, 41, 42]. The epigenetic alterations we identified, particularly in regulatory genes like *IL10* and *FOXP3*, may define a subgroup of patients with broader immune dysfunction. Prior work has shown that such patients often have heightened systemic inflammation and an increased burden of extra‐pelvic symptoms [43, 44]. Future research should assess whether these epigenetic signatures are enriched in patients with comorbid autoimmune conditions (e.g., lupus, rheumatoid arthritis), which would clarify links between immune dysregulation, epigenetic control, and the widespread clinical manifestations seen in UCPPS. These studies would address underlying mechanisms and clarify patient phenotypes based on the mechanism of action for future clinical trial designs in UCPPS [45].

Several limitations should be considered when interpreting these study findings. The patient cohort represents a heterogeneous clinical population, and VB3 sediments and PBMC preparations contain multiple cell types, such that the transcript and methylation profiles likely reflect composite immune signatures rather than cell‐type‐specific regulation. In addition, direct correlations between methylation status and functional gene expression were not assessed within the same cellular populations. Finally, the animal studies were performed in inflammatory pain models that may not fully capture the complexity of human CP/CPPS. Future studies in larger, well‐characterized patient cohorts and complementary mechanistic models will be required to more precisely define the role of epigenetic regulation in CP/CPPS pathogenesis.

## Author Contributions

**Praveen Thumbikat:** directed study, analyzed data, prepared manuscript. **Goutham Pattabiraman:** performed study and analyzed data. **Farzaneh Sharifzad:** performed study and analyzed data. **Catherine V. Osborn:** performed study and analyzed data. **Stephen F. Murphy:** performed study and analyzed data. **Yongyong Yang:** performed study and analyzed data. **Zhiqiang Liu:** performed study and analyzed data. **Qi Cao:** performed study, reviewed manuscript. **Anthony J. Schaeffer:** performed study, reviewed manuscript.

## Ethics Statement

The Northwestern University Institutional Review Board (Panel D) approved the protocols (IRB STU00030121, STU00202831, and STU00215831). All animal experiment procedures were approved by the Northwestern University Animal Care and Use Committee.

## Consent

All participants provided written informed consent.

## Conflicts of Interest

The authors declare no conflicts of interest.

## Data Availability

Data developed during this study are available on request. Clinical data made available, upon request, will follow established protocol guidance by Northwestern University IRB.
